# LRP-1 Promotes Colon Cancer Cell Proliferation in 3D Collagen Matrices by Mediating DDR1 Endocytosis

**DOI:** 10.3389/fcell.2020.00412

**Published:** 2020-06-03

**Authors:** Cao Cuong Le, Amar Bennasroune, Guillaume Collin, Cathy Hachet, Véronique Lehrter, Damien Rioult, Stéphane Dedieu, Hamid Morjani, Aline Appert-Collin

**Affiliations:** ^1^Université de Reims Champagne-Ardenne, Reims, France; ^2^CNRS UMR 7369, Matrice Extracellulaire et Dynamique Cellulaire, MEDyC, Reims, France; ^3^Unité BioSpecT, EA7506, Reims, France; ^4^Plateau Technique Mobile de Cytométrie Environnementale MOBICYTE, URCA/INERIS, Reims Champagne-Ardenne University (URCA), Reims, France

**Keywords:** LRP-1, DDR1, colon cancer cell, proliferation, 3D collagen matrix

## Abstract

Low density lipoprotein receptor related protein-1 (LRP-1) is a large ubiquitous endocytic receptor mediating the clearance of various molecules from the extracellular matrix. Several studies have shown that LRP-1 plays crucial roles during tumorigenesis functioning as a main signal pathway regulator, especially by interacting with other cell-surface receptors. Discoïdin Domain Receptors (DDRs), type I collagen receptors with tyrosine kinase activity, have previously been associated with tumor invasion and aggressiveness in diverse tumor environments. Here, we addressed whether it could exist functional interplays between LRP-1 and DDR1 to control colon carcinoma cell behavior in three-dimensional (3D) collagen matrices. We found that LRP-1 established tight molecular connections with DDR1 at the plasma membrane in colon cancer cells. In this tumor context, we provide evidence that LRP-1 regulates by endocytosis the cell surface levels of DDR1 expression. The LRP-1 mediated endocytosis of DDR1 increased cell proliferation by promoting cell cycle progression into S phase and decreasing apoptosis. In this study, we identified a new molecular way that controls the cell-surface expression of DDR1 and consequently the colon carcinoma cell proliferation and apoptosis and highlighted an additional mechanism by which LRP-1 carries out its sensor activity of the tumor microenvironment.

## Introduction

The low-density lipoprotein (LDL) receptor-related protein (LRP) superfamily contains twelve transmembrane proteins participating in a wide range of physiopathological processes (Emonard et al., 2014; Pohlkamp et al., 2017). Belonging to this family, LRP-1 is widely expressed in a large variety of tissues and exhibits functionalities in controlling key biological processes such as pericellular protease activities and extracellular matrix (ECM) function. This protein consists of a large functional endocytic receptor firstly synthesized as a 600-kDa precursor cleaved to an extracellular ligand-binding subunit of 515 kDa and a transmembrane 85 kDa part containing a 100 amino acids cytosolic tail. LRP-1-mediated endocytosis is tightly coupled to regulation of signaling pathways (Muratoglu et al., 2010; Mantuano et al., 2013; Kang et al., 2014). LRP-1 can indeed regulate mitogen-activated protein kinases (MAPK) as well as the survival-associated PI3K/Akt signaling pathway (Fuentealba et al., 2009; Langlois et al., 2010; Roura et al., 2014). LRP-1-dependent endocytosis and signaling-related events have been shown to play critical roles in severe pathologies including both Parkinson’s and Alzheimer’s diseases, metabolism dysfunction and cancer. Regarding tumor growth and metastasis, the molecular contribution of LRP-1 remains misunderstood and be highly dependent of the tumor microenvironment. Although LRP-1 expression and its role in cancer hallmarks are now well referenced in glioma (Boyé et al., 2017), melanoma (Salama et al., 2019), thyroid (Perrot et al., 2012; Appert-Collin et al., 2017; Theret et al., 2017), and breast carcinoma (Beaujouin et al., 2010; Tian et al., 2019), little is known about LRP-1 functionalities in colorectal carcinoma (CRC). LRP-1B, a member of LDL-R family highly homologous to LRP-1, is downregulated in the colon cancer tissues and inhibits the growth, migration and metastasis of colon cancer cells (Wang et al., 2017). Previous studies based on few colon adenocarcinoma samples have shown a frequent loss of LRP-1 immunohistochemical expression in adenocarcinomatous cells (Obermeyer et al., 2007; Toquet et al., 2007). A recent clinical study from our team demonstrated that LRP-1 expression was significantly lower in colon adenocarcinoma cells compared to colon epithelial cells and stromal cells and that this decrease in LRP-1 expression is associated with worse patient outcomes (Boulagnon-Rombi et al., 2018). Moreover, LRP-1 mutations have been reported in patients with liver metastasis (Wu et al., 2019). In the light of these data, the role of LRP-1 in CRC remains poorly understood and deserves to be further studied, especially to gain molecular insights.

Type I collagen is one of the main components of the cellular microenvironment in many mammalian tissues and plays a crucial role in tumor progression in several solid tumors, particularly in CRC (Brabletz et al., 2004). This protein is highly expressed in CRC with infiltrative growth phenotype (Oku et al., 2008). Two cellular groups of membrane receptors can interact with type I collagen, β1 integrin heterodimers and discoidin domain receptors (DDRs). DDR1 and DDR2 are the only receptors of collagen harboring a tyrosine kinase function (Leitinger, 2003; Abdulhussein et al., 2004; Rammal et al., 2016). DDR1 is activated by most collagen types, including I and IV, whereas DDR2 is only activated by fibrillary collagens. Upon collagen binding, activation of DDR1 and DDR2 are associated with a slow and sustained self-phosphorylation in comparison to other tyrosine kinase receptors (Shrivastava et al., 1997; Vogel et al., 1997). Indeed, tyrosine residues of DDR receptors are phosphorylated after 2 h and can be maintained for several hours. DDR1 expression has been associated with an increase in tumor invasion and aggressiveness of many human tumors, including esophageal cancer (Nemoto et al., 1997), gastric cancer (Xie et al., 2016), glioma (Yamanaka et al., 2006), breast cancer (Malaguarnera et al., 2015), and lung cancer (Xiao et al., 2015). The role of DDR1 in the regulation of tumor cell proliferation and apoptosis remains poorly documented and somewhat controversial. In breast cancer, DDR1 activates the insulin-like growth factor I receptor (IGF-1R) to support several IGF-1R-mediated biological responses such as cell proliferation (Malaguarnera et al., 2015). In lung cancer cells, DDR1 knockdown has been reported to decrease ERK and Akt phosphorylation leading to a downregulation of cell proliferation suggesting a role of DDR1 autophosphorylation triggered by collagen IV binding in lung cancer progression (Xiao et al., 2015). However, other studies have demonstrated that, in breast carcinomas, DDR1 promotes apoptosis through induction of pro-apoptotic protein BIK1 (Assent et al., 2015; Saby et al., 2018, 2019). In the case of CRC, recent studies have shown that nilotinib, a specific inhibitor of DDR1 phosphorylation, strongly reduced DDR1-mediated CRC cell invasion and metastasis in mouse models (Jeitany et al., 2018), and that the use of antibody-drug conjugates targeting DDR1 exhibits antitumor effects in a mouse model of CRC (Tao et al., 2019). These works have been carried out on CRC cells harboring invasive-like phenotype. Concerning the non-invasive epithelial-like carcinoma cells, a previous study reported a down-regulation of cell proliferation using 3D matrix, but the role of DDR1 in such a process was not explored (Luca et al., 2013).

In the present work, we investigated whether LRP-1 may control DDR1 expression at the plasma membrane in non-invasive CRC and influence its ability to regulate tumor cell proliferation upon its activation by type I collagen. Our data demonstrate for the first time that LRP-1 can induce endocytosis of DDR1 in CRC, thus decreasing the ability of the 3D collagen matrix/DDR1 axis to inhibit tumor cell proliferation.

## Materials and Methods

### Cell Lines

LS174T (Duke’s type B), HT-29 and RKO cell lines were purchased from American Type Culture Collection (ATCC, Rockville, MD, United States). LS174T, HT-29 and RKO cells were grown in Eagle’s Minimum Essential Medium (EMEM) (ATCC 30-2003) or in Dulbecco’s Modified Eagle Medium (DMEM) with high glucose (4.5 g/L) (Thermo scientific), respectively. Culture media were supplemented with 10% (v/v) fetal bovine serum (FBS) (Dutscher, France) and 1% penicillin-streptomycin (v/v, Invitrogen, Cergy-Pontoise, France). Cultures were maintained at 37°C in a humidified atmosphere containing 5% CO2 (v/v). Cells were routinely passaged at preconfluency using 0.05% trypsin, 0.53 mM EDTA (Invitrogen, 25300) and screened for the absence of mycoplasma using PCR methods.

### Vectors, Transfection and Infection

DDR1-GFP overexpression was performed using pLVX-CMV-DDR1-GFP construct which was a generous gift from Frederic Saltel (INSERM, UMR1053, BaRITOn Bordeaux Research in Translational Oncology, Bordeaux, France). DDR1-GFP lentiviral particles were generated by transient co-transfection of 293T with pCMV ΔR8.91 (gag-pol) and phCMVG-VSVG (env) expression constructs using the FuGene 6 transfection reagent (Promega) according to manufacturer’s recommendations. Three days after transfection, the supernatant containing lentiviruses was collected, filtered through 0.45 μm filter, mixed with fresh medium (1 of 4) and hexadimethrine bromide at 8 μg/ml (Sigma) and used to infect HT-29 recipient cells. GFP control cells were processed in the same way. Infected cells were selected using puromycin (Invitrogen) at 3 μg/ml. LRP-1 knock-down was achieved using shRNA sequences previously described (Dedieu et al., 2008) that were purchased from Sigma. shRNA LRP-1 lentiviral particles were produced in 293T cells using FuGene 6 transfection reagent (Promega) and used to infect HT-29 recipient cells as described above. HT-29 cells expressing control shRNA were generated in the same way. Infected cells were selected using puromycin (Invitrogen) at 3 μg/ml.

### 2D and 3D Cell Culture

Fibrillar native type I collagen was extracted from tail tendons of 2-month-old rats and prepared as already described (Saby et al., 2016). For 2D cell cultures, each well was coated with 5 μg/cm^2^ of collagen solubilized in 0.018 M acetic acid. Coated substrates were dried overnight at room temperature (RT) under sterile conditions. Thereafter, wells were washed two times with PBS (Invitrogen) before cell plating. In cell proliferation studies, cells were seeded on the coated surfaces at a density of 15 × 10^3^ cells/well or 5 × 10^3^ cells/0.33 cm^2^ (24 well plates). In other studies, cell density was adjusted depending on the confluence. To quantify cell proliferation, after 5 days, cells were detached by trypsin and counted by phase-contrast microscopy (multiple-repeated counting for each condition). Each condition was done in triplicate and repeated in at least three biological experiments. For 3D culture, cells were seeded at a final density of 15 × 10^3^ cells/mL. For that, 3 × 10^4^ cells were resuspended in 100 μl of FBS and mixed with a solution containing 100 μl of 10X DMEM culture medium for HT-29 cells or EMEM for LS174T cells, 100 μl NaHCO3 (0.44 M), 90 μl NaOH 0.1 M, 10 μl sodium pyruvate, 10 μL Ampicillin + Streptomycin, (and 10 μl glutamine 200 mM for MEM culture medium), the premix is adjusted to 500 μl with sterile ultrapure water. After that, the mix containing cells is gently homogenized with 500 μl of collagen 3 mg/ml to finalize the collagen-based medium. Then, 1 ml/well of this pre-solidified medium was deposited in 24-well plates, and collagen gel solidification was performed at 37°C during 30 min. Finally, 1 ml of complete culture medium was added on top of each gel and the plates were incubated at 37°C. Covering medium is changed every 2 days. After 3 or 5 days, the covering medium was removed, and gels were digested with 2 mg/ml collagenase P (Roche). After collecting the cells from digested gel, cells were dissociated by tryspin and viable cell number was determined by phase contrast microscopy using Kova Glasstic Slides (Kova International Inc., Garden Grove, CA, United States).

### Antibodies and Recombinant Proteins

Anti-LRP1 β-chain antibody (clone EPR3724) was purchased from Abcam (Cambridge, United Kingdom). Rabbit monoclonal antibodies against DDR1 (D1G6), phospho-DDR1 (Tyr792, 4G10), GFP (D5.1), and GAPDH (14C10) were purchased from cell signaling. Rabbit monoclonal antibodies against DDR2 were purchased from R&D systems (Lille, France). IgGs used as a negative control for immunoprecipitation and cell treatments were purchased from Santa Cruz Biotechnology (Heidelberg, Germany). Blocking LRP-1 polyclonal antibody (R2629) was a generous gift from Dr. D. K. Strickland (Department of Surgery, University of Maryland School of Medicine, Baltimore, MD, United States) (Mikhailenko et al., 2001). Histidine-tagged RAP (Receptor-associated protein) was purified as previously described (Dedieu et al., 2008).

### RNA Isolation and qPCR

Total mRNAs were extracted using TRIzol reagent (Thermofisher), isolated from other cellular materials by chloroform/isoamyl alcohol (24:1) precipitation before centrifugation (12,000 × *g*, 4°C, 15 min), as previously described (Theret et al., 2017). 250 ng total mRNAs were reverse-transcribed using VERSO cDNA kit (Thermofisher) according to the manufacturer instructions. Real-time PCR was then performed using an Absolute SYBR Green Rox mix (Thermofisher) and a CFX 96 real time PCR detection system (Bio-Rad, Hercules, CA, United States). The cycle threshold (Ct) values were recorded using Bio-Rad CFX Manager 3.0 software (Bio-Rad) (Le Cigne et al., 2016). Results are expressed as 1/DCt. DCt corresponds to the difference between Ct of the sequence of interest and Ct of our reference sequences (RS18 and RPL32) (Scandolera et al., 2015). PCR primers were synthesized by Eurogentec (Liege, Belgium) as follow (5′-3′): for LRP1: GCTATCGACGCCCCTAAGAC and CGCCAGCCCTTTGAGATACA; for DDR1: ACTTTGGCAT GAGCCGGAAC and ACGTCACTCGCAGTCGTGAAC; for RS18: GCAGAATCCACGCCAGTACAA and GCCAGTGGTC TTGGTGTGCT; for RPL32: CATTGGTTATGGAAG CAACAAA and TTCTTGGAGGAAACATTGTGAG.

### Total Protein Extraction and Immunoblotting

Cells were seeded in 3D type I collagen matrix for 5 days, then were harvested from digested matrices using collagenase P (2 mg/ml), washed twice with PBS, and lysed. The cells were then pelleted by centrifuging at 1000 rpm for 5 min. Whole-cell extracts were lysed in RIPA buffer (Thermofisher), sonicated and then incubated on ice. Cell lysates were collected after a centrifugation at 14000 rpm and 4°C for 15 min. Protein concentration was quantified by BCA assay (Thermofisher). Proteins were separated by sodium dodecyl sulfate-polyacrylamide gel electrophoresis (SDS-PAGE) and transferred onto a nitrocellulose membrane (Amersham Biosciences, Little Chalfont, United Kingdom). Membranes were blocked with 5% skimmed milk (m/v) in Tris buffered saline (0.02 M Tris–HCl, 0.137 M NaCl, pH 7.4), supplemented with 1% Tween 20 (v/v) at RT for 1 h. Blocked membranes were incubated with antibodies against LRP-1 β-chain (EPR3724), DDR1 (D1G6) and GAPDH (14C10) overnight at 4°C under gentle agitation. Finally, membranes were incubated with corresponding peroxidase conjugated secondary antibody at RT. Chemiluminescent reactions were revealed by using ECL Prime Kit (GE Healthcare, Orsay, France), signal was detected by the Odyssey-FC system (Licor, Lincoln, NE, United States).

### Cell Surface Protein Isolation

The cells were treated with or without 500 nM RAP for 1 h, washed twice with PBS before suffering a biotinylation with 0.5 μg/mL of EZ-Link sulfo-NHS-LC-biotin (Thermofisher) in cold PBS. After three washes, biotinylated cells were incubated with 100 mM glycine at 4°C during 30 min to limit nonspecific binding. Cells were washed three times with PBS before protein extraction. Cells were scrapped in cold lysis buffer (10 mM Tris HCl, pH 7.5, 150 mM NaCl, 2 mM Na3VO4, 5 mM EDTA, 1% Triton X-100, supplemented with protease inhibitor cocktail), followed by a quick sonication on ice. Cell extracts were pelleted at 10,000 g (20 min, 4°C) before protein quantification. Solubilized biotinylated proteins (200 μg) were then affinity purified using 40 μL of streptavidin-agarose beads (GE Healthcare), overnight at 4°C under gentle agitation. After washes with lysis buffer, Laemmli buffer was added and samples were heated at 100°C for 5 min and resolved by SDS-PAGE followed by immunoblotting analysis.

### Endocytosis Assay

Endocytosis assays were adjusted from validated method (Theret et al., 2017). Cell-surface proteins were labeled using 0.5 μg/mL of EZ-Link sulfo-NHS-LC-biotin (Thermofisher) in cold PBS at 4°C for 30 min. After washes with PBS, cells were incubated with 100 mM glycine at 4°C for 15 min. Nonspecific binding and free biotin were discarded by warm PBS washes before addition of warm medium supplemented with 10% FBS. Cells were treated with 500 nM RAP at 37°C for 1 h to antagonize endocytosis function of LRP-1. Cells were then quickly placed on ice to block internalization activities. After three washes with PBS, cells were incubated with 50 mM glutathione in cold buffer (75 mM NaCl, 75 mM NaOH, 1 mM MgCl2, 0.1 mM CaCl2, 10 mM EDTA, pH 8.6) at 4°C for 30 min to remove remaining biotin at the cell surface. To evaluate the total amount of surface biotinylation, one culture dish was kept on ice after biotin labeling and preserved from glutathione treatment. The efficiency of glutathione efficacy at the cell surface was controlled to be over 90%. Whole-cells extracts were prepared as described above. Internalized DDR1 was determined from 350 μg of cell lysate by adding 40 μL of streptavidin-agarose beads (GE Healthcare), incubating overnight at 4°C under gentle agitation and using DDR1 antibody through immunoblotting, as described above.

### Immunoprecipitation

Whole cell extracts from HT-29^*DDR*1–*GFP*^ were performed as described in a previous study (Theret et al., 2017). Whole cell lysates were subjected to immunoprecipitation using anti-LRP-1 (EPR3724), anti-DDR1 (D1G6) antibodies or nonspecific IgGs at 4°C for 12 h, bound to protein G sepharose beads (GE Healthcare) at 4°C for 2 h and finally washed three times with cold lysis buffer followed by a protein denaturation step at 100°C for 5 min. After that, the samples were centrifuged at 10000 rpm for 1 min, supernatants were then subjected to a western blot analysis using anti-LRP-1 β-chain (clone EFR3724), anti-DDR1 (D1D6), and anti-GFP antibodies.

### DDR1 Phosphorylation Analysis

HT-29 and HT-29 overexpressing DDR1-GFP (HT-29^*DDR*1–*GFP*^) cells were cultured in 3D type I collagen matrices with or without 50 nM nilotinib treatment for 16 h. Matrices were digested before undergoing a standard procedure for total protein extraction in 3D (Saby et al., 2016). Then, 300 μg of whole-cell extracts were immunoprecipitated with anti-DDR1 (D1D6), as described above. The proteins were separated by SDS-PAGE and the immunoprecipitates were blotted with anti-phosphotyrosine antibody, clone 4G10 (Millipore, 05-321). The blots were then stripped using a stripping buffer (200 mM glycine, 1% SDS, 0.02% sodium azide, pH 2.5) and re-probed with anti-DDR1 antibody.

### Cell Cycle

Double thymidine block procedure was adapted from an established protocol (Chen and Deng, 2018). Specifically, HT-29 and HT-29^*DDR*1–*GFP*^ cells were cultured in medium supplemented with 2 mM thymidine for 18 h then switched to thymidine-free medium for 9 h. After two washes with PBS, cells were cultured again in medium supplemented with 2 mM thymidine for 15 h. Cells were released by washing twice with PBS before trypsinization. The synchronized cells were then seeded into 3D type I collagen matrices with or without 1 μM RAP treatment for 24 h. Collagen matrices were further digested to harvest cultured cells. Lastly, cells were washed twice with PBS and stained with nuclear isolation medium-4,6-diamidino-2-phenylindole dihydrochloride named NIM-DAPI (NPE Systems, Pembroke Pines, FL, United States) at RT for 5 min. The samples were analyzed with an Accuri-C6 Special Order Product (BD Bioscience) by acquisition of 20000 events. Analysis was performed with an excitation wavelength of 375 nm and fluorescence detection at 427 ± 10 nm.

### Apoptosis Assay

HT-29 and HT-29^*DDR*1–*GFP*^ cells were cultured in 3D type I collagen matrices with or without 1 μM RAP treatment for 3 days. The culture medium was replaced every 2 days by fresh complete DMEM medium with or without 1 μM RAP. After 5 days, cells were harvested as described above. Harvested cells were washed with PBS before suffering a quick trypsinization. The single cells were then incubated with Annexin V-iFluor 647 Apoptosis solution (Abcam, United Kingdom), supplemented with propidium iodide (Sigma-Aldrich). The incubation was carried out at RT for 30 min. Apoptosis assays were performed using flow cytometer, FL4 channel (BD Biosciences, San Jose, CA, United States).

### Immunofluorescence

HT-29^*DDR*1–*GFP*^ cells were seeded onto collagen-coated glass slides for 48 h at 37°C and then fixed in PBS containing 4% paraformaldehyde for 15 min at RT. After three washes with PBS, cells were incubated for 30 min in PBS containing 1% bovine serum albumin and then incubated overnight at 4°C with GFP primary antibodies. Then, after five washes with PBS, cells were incubated with secondary antibodies conjugated to Alexa Fluor 488 (1/1000) during 1 h at RT. DAPI was added during washes. Slides were incubated with mounting medium. Immunofluorescence-labeled cell preparations were analyzed using a Zeiss LSM 710 confocal laser scanning microscope with the 63× oil-immersion objective Zeiss operating system (Carl Zeiss MicroImaging GmbH, Deutschland).

### Data Analysis

All statistical results were analyzed from at least three independent experiments. Data were represented as the standard deviation (SD) using Graphpad Prism software. Student’s *t*-test and ANOVA test were used for statistical analysis. Immunoblotting images were analyzed by ImageJ software.

## Results

### LRP-1 Inhibition Decreases Colon Carcinoma Cell Proliferation Only in 3D Collagen Matrices

**FIGURE 1:**
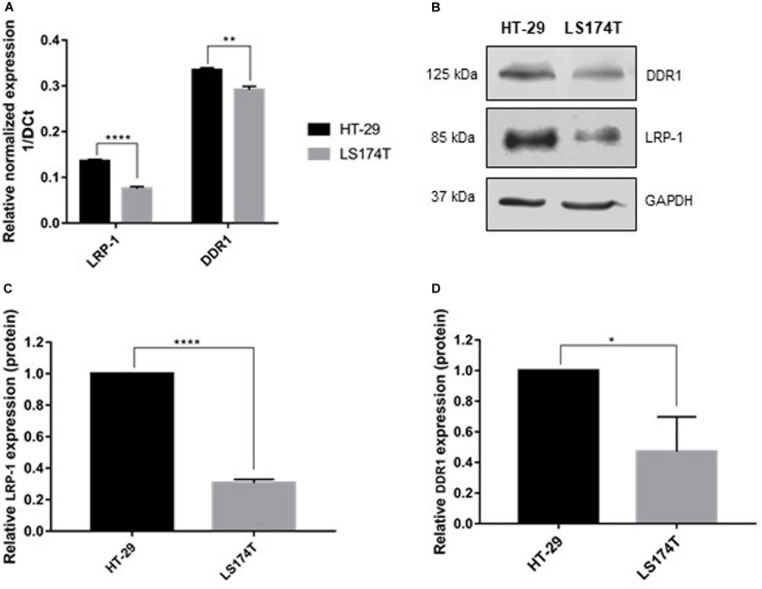
Molecular characterization of colorectal carcinoma cell lines. **(A)** Transcriptional level of LRP-1 and DDR1 were assessed using RTqPCR. LRP-1 and DDR1 mRNA expression levels in HT-29 (black boxes) and LS174T (gray boxes) were normalized with both RPL32 and RS18 mRNA expression. **(B)** Whole cell extracts from HT-29 and LS174T cells were analyzed by SDS PAGE followed by western blotting using anti-DDR1, anti-LRP-1 and anti-GAPDH antibodies. Graphical representations of LRP-1 **(C)** and DDR1 **(D)** expression at protein level as normalized with GAPDH. All experiments were performed in three biological replicates. Plots are presented as the mean SD, **p < 0.01; ****p < 0.0001, *n* = 3, two sample *t*-test. *p = 0.01.

To study the involvement of LRP-1 and DDR1 in the regulation of colon tumor cell proliferation by 3D collagen matrix, the endogenous expression level of LRP-1 and DDR1 were analyzed by both RT-qPCR and immunoblotting in LS174T and HT-29 cells (Figure 1). Results showed that the expression of the two receptors at the mRNA and protein levels in HT-29 cells are higher than in LS174T cells. It should be noted that DDR2 is not expressed in these two cells lines (data not shown).

**FIGURE 2:**
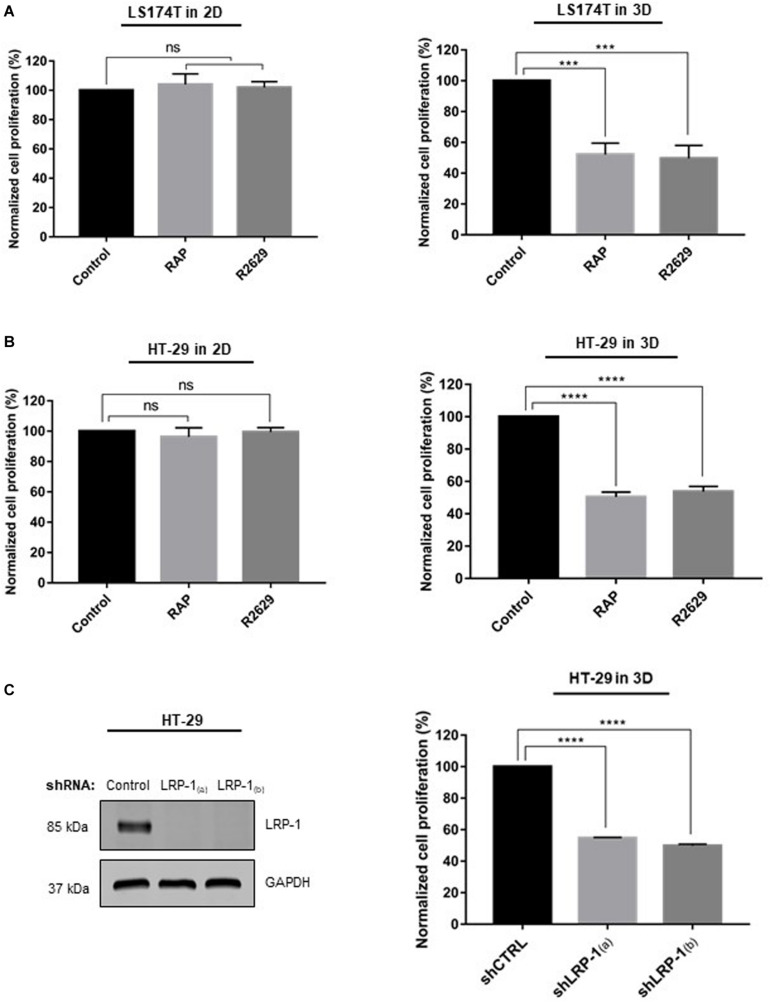
Effect of LRP-1 antagonists and LRP-1 knockdown on colorectal cancer cell proliferation. LS174T **(A)** and HT-29 **(B)** cells were cultured in 2D type I collagen coating (left panels) or 3D type I collagen matrices (right panels) without (black boxes) or with RAP (500 nM, light gray boxes) or R2629 (2.5 μg/mL, dark gray boxes) treatment. After 5 days of culture, cell growth indices were assessed using at least three separate sets of culture, all conditions were repeated at least three times. **(C)** HT-29 cells were transduced with lentivirus encoding non-silencing shRNA (shCTRL) or shRNA targeting LRP-1 [shLRP1_(*a*)_ and shLRP1_(*b*)_] (right panel). Whole-cell extracts from each clonal cell were submitted to immunoblot analysis using anti-LRP-1 antibody (5A6). GAPDH expression level served as a loading control. shCTRL (black boxes) and shLRP-1_(*a*)_ or shLRP-1_(*b*)_ (gray boxes) HT-29 cells were seeded in 3D type I collagen matrix (left panel) during 5 days with or without RAP and R2629 treatment. Cell growth was evaluated by at least three separate experiments, each done in triplicate. The data are presented as the mean SD. ***p < 0.001; ****p = 0.0001; ns: not significant, One-way ANOVA test using Dunnett’s multiple comparisons.

We then examined the effect of LRP-1 inhibition on HT-29 and LS174T cell proliferation in 2D and in 3D collagen matrices. For this purpose, we compared the cell proliferation after 5 days of culture in the presence or absence of RAP (receptor associated protein), the LRP-1 antagonist, or its blocking antibody (R2629). As shown in Figures 2A,B, in both cell lines, treatment by RAP or R2629 did not modify cell proliferation in 2D collagen coating. By contrast, 3D-cell proliferation was decreased by about 50% in each cell line when using RAP or R2629 treatment whereas IgG treatment has no effect (Supplementary Figure S1). To focus on the role of LRP-1 in the regulation of cell proliferation in 3D collagen matrices, an RNA interference strategy against LRP-1 was performed in HT-29 cells. Two different cell lines that stably overexpressed a specific shRNA for LRP-1 [shLRP-1_(a)_ and shLRP-1_(b)_] were selected, and a control cell line was established after infection with control shRNA (shCTRL). The endogenous level of LRP-1 was assessed by both RT-qPCR and immunoblotting (Figure 2C, left panel). As expected, infection with lentiviruses expressing shCTRL had no effect on the LRP-1 expression level while LRP-1-specific shRNA was able to efficiently knock-down the expression of LRP-1 at the mRNA level (data not shown) as well as at the protein level by about 90%. The same inhibition was observed for both shLRP-1 cell lines (Figure 2C, left panel). As shown in Figure 2C (right panel), proliferation of LRP-1-silenced cancer cells was decreased by about 50% in 3D collagen matrices, whereas no effect of LRP-1 silencing was observed in 2D (data not shown). Taken together, these data indicate that LRP-1 sustains colon cancer cell proliferation, and that this process occurs only in a 3D collagen environment. Similar study on cell proliferation have been conducted using RKO colon carcinoma cells that do not express DDR1 (Supplementary Figure S2A). Results demonstrated that LRP-1 inhibition by RAP did not modify RKO cell proliferation both in 2D and in 3D matrices, suggesting that LRP-1 supports CRC proliferation in a DDR1 dependent manner (Supplementary Figure S2B).

### LRP-1 and DDR1 Coexist Within the Same Molecular Complexes

**FIGURE 3:**
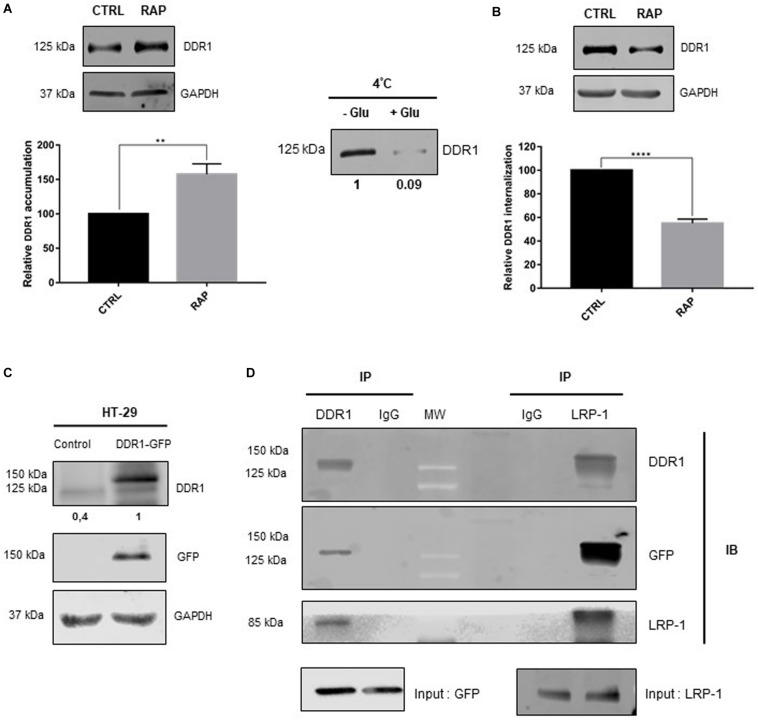
RAP treatment inhibits DDR1 endocytosis and led to its accumulation at the plasma membrane. **(A)** Plasma membrane extracts from cell surface biotinylated proteins were obtained from HT-29 cells treated or not with RAP (1 μM, 1 h). Immunoblot analysis was performed using anti-DDR1 antibodies. Expression level of GAPDH in the intracellular fraction served as a loading control and for normalization. Three independent experiments were conducted, the data is represented as the mean SD. **p < 0.005, two sample *t*-test. **(B)** HT-29 cells were treated with/without RAP (1 μM) for 1 h. Plasma membrane proteins were biotinylated and endocytosis assay was carried out as reported in the experimental procedure section. DDR1 internalization was quantified by immunoblotting using DDR1 antibody (right panels including graph, ****p < 0.0001, two sample *t*-test). Left panel (4°C) serves to control DDR1 binding to the cell surface (-Glut, without glutathione) and glutathione efficacy for biotin stripping (+ glut, with glutathione). **(C)** Whole-cell extracts were obtained from HT-29 cells overexpressing GFP (control) or DDR1-GFP. Immunoblot analysis was performed using anti-DDR1 and anti-GFP antibodies and GAPDH served as a loading control. LRP-1 **(D)** or DDR1 containing complexes were immunoprecipitated (IP) from DDR1-GFP overexpressing HT-29 cells whole-cell extracts by using anti-LRP-1 (clone EPR3724) or anti-DDR1 (D1G6) monoclonal antibody, respectively. Immunocomplexes were then subjected to SDS-PAGE and immunoblotted (IB) by using specific antibodies for LRP-1, DDR1, and GFP.

Since LRP-1 had a positive effect on cell proliferation only in 3D collagen environment by LRP-1, we hypothesized that LRP-1 could interact with DDR1, one of the key collagen membrane receptors, to induce its endocytosis. To validate this hypothesis, we first evaluated whether LRP-1 may influence the DDR1 amount at the plasma membrane of HT-29 cells. After cell-surface protein labeling with the membrane-impermeable biotinylation reagent sulfo-NHS-LC-biotin, biotinylated proteins were selectively recovered from cell extracts by streptavidin affinity precipitation, and DDR1 was detected in the affinity precipitates by immunoblot analysis. After RAP treatment, DDR1 was found to accumulate at the plasma membrane fraction (Figure 3A), suggesting that LRP-1 mediates DDR1 internalization. Thus, we investigated DDR1 uptake by using a previously validated endocytosis assay (Theret et al., 2017). This method requires labeling of cell surface proteins using the non-membrane permeating sulfo-NHS-LC-biotin at 4°C, then moving to a permissive temperature for endocytosis (37°C). Cell surface protein biotinylation as well as efficiency of biotin stripping with glutathione were controlled (Figure 3B, left panel). As shown in Figure 3B (right panel), DDR1 internalization was decreased by about 40% when LRP-1-mediated endocytosis was antagonized by RAP treatment. To test whether LRP-1 and DDR1 may participate in a common biomolecular complex, coimmunoprecipitation experiments were carried out in DDR1 overexpressing HT-29 cells (HT-29^*DDR*1–*GFP*^). As shown in Figure 3C, HT-29^*DDR*1–*GFP*^ expressed a high level of recombinant DDR1-GFP. Our data clearly demonstrated that DDR1 was coimmunoprecipitated with LRP-1. Reverse immunoprecipitation experiments with anti-DDR1 were also performed using the same cell lysates and confirmed that LRP-1 and DDR1 were detected in the same molecular complexes in colon carcinomas (Figure 3D).

### LRP-1 Promotes HT-29 Proliferation in a DDR1 Dependent Fashion

**FIGURE 4:**
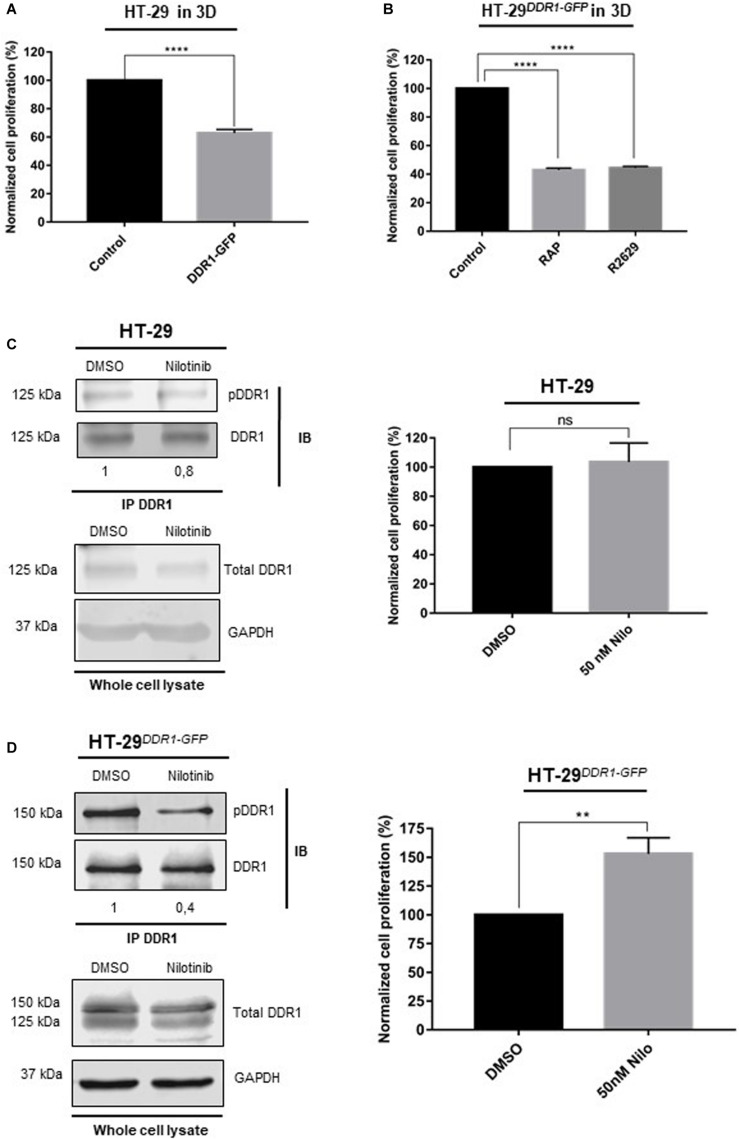
DDR1 down-regulates colorectal cancer cell proliferation in 3D collagen matrix. **(A)** Wild-type HT-29 were cultured in 3D type I collagen matrix for 5 days, then cell proliferation was evaluated by three independent experiments. The data are represented as the mean SD, ****p < 0.0001, two sample *t*-test. **(B)** HT-29^*DDR*1–*GFP*^ cells were seeded in 3D type I collagen matrix during 5 days with/without RAP or LRP-1 blocking antibodies (R2629). Cell proliferation was then evaluated by at least 3 separate sets of culture, the data are presented as the mean SD and compared to untreated cells. ****p = 0.0001, One-way ANOVA test using Dunnett’s multiple comparisons. HT-29 **(C)** and HT-29^*DDR*1–*GFP*^ cells **(D)** were seeded in 3D type I collagen matrix and cultured with 50 nM nilotinib or DMSO (that served as a control) for 5 days. Left panels: DDR1 containing complexes were immunoprecipitated (IP) whole-cell extracts by using an anti-DDR1 monoclonal antibody (D1G6). Immunocomplexes were then subjected to SDS-PAGE and immunoblotted (IB) by using anti-DDR1 (D1G6) and anti-phospho-DDR1 (Tyr792, 4G10). Numbers under the immunoblots indicate the fold change ratio (pDDR1/DDR1), as compared to DMSO-treated cells that serve as the reference (*n* = 3). The bottom panel indicates the expression of DDR1 and GAPDH in whole cell lysates and served as a control. Right panels: cell proliferation was evaluated by three independent experiments, the data are presented as the mean SD. **p < 0.005; ns: not significant, two sample *t*-test.

Previous reports demonstrated that cancer cell growth was downregulated by 3D type I collagen matrix in epithelial-like breast carcinoma cells and that this was dependent on activation of DDR1 by collagen (Assent et al., 2015; Saby et al., 2018). Considering that LRP-1 induced HT29 cell proliferation in 3D collagen matrices (Figure 2) and drives endocytosis of DDR1 (Figure 3), we assume that the cell-surface expression level of DDR1 may constitute a key parameter to control growth and survival of colon cancer cells. To address this hypothesis, we compared the cell proliferation of HT-29^*DDR*1–*GFP*^ and control counterparts. As expected, overexpression of DDR1 led to decreased cell proliferation in collagen 3D matrices by about 40%, compared to control cells (Figure 4A). Furthermore, cell proliferation in collagen 3D matrices was decreased by about 60% under RAP or R2629 antibody treatments in HT-29 cells overexpressing DDR1-GFP (Figure 4B). Interestingly, the proliferative inhibition under LRP-1 antagonization was more important when DDR1 was overexpressed.

### DDR1 Activity Is Necessary to Induce Cell Proliferation in HT-29^*DDR*1–*GFP*^ Cells

Since DDR1 phosphorylation could be responsible of growth inhibition (Saby et al., 2018), we evaluated the impact of nilotinib (50 nM), a receptor tyrosine kinase inhibitor with high potency against DDR1, on colon carcinoma cell proliferation using both control (Figure 4C) and HT-29^*DDR*1–*GFP*^ (Figure 4D) cells. Nilotinib treatment had no effect on cell proliferation in control cells (Figure 4C, right panel) whereas carcinoma cell proliferation in DDR1-overexpressing cells was increased after nilotinib treatment (Figure 4D, right panel). Consistently, DDR1 phosphorylation was drastically inhibited upon nilotinib treatment in HT-29^*DDR*1–*GFP*^ cells (Figure 4D, left panel). Taken together, these data suggest that collagen-induced cell growth inhibition relies on DDR1 phosphorylation.

### LRP-1 Inhibition Induces Cells Cycle Arrest in the G0/G1 Phase

**FIGURE 5:**
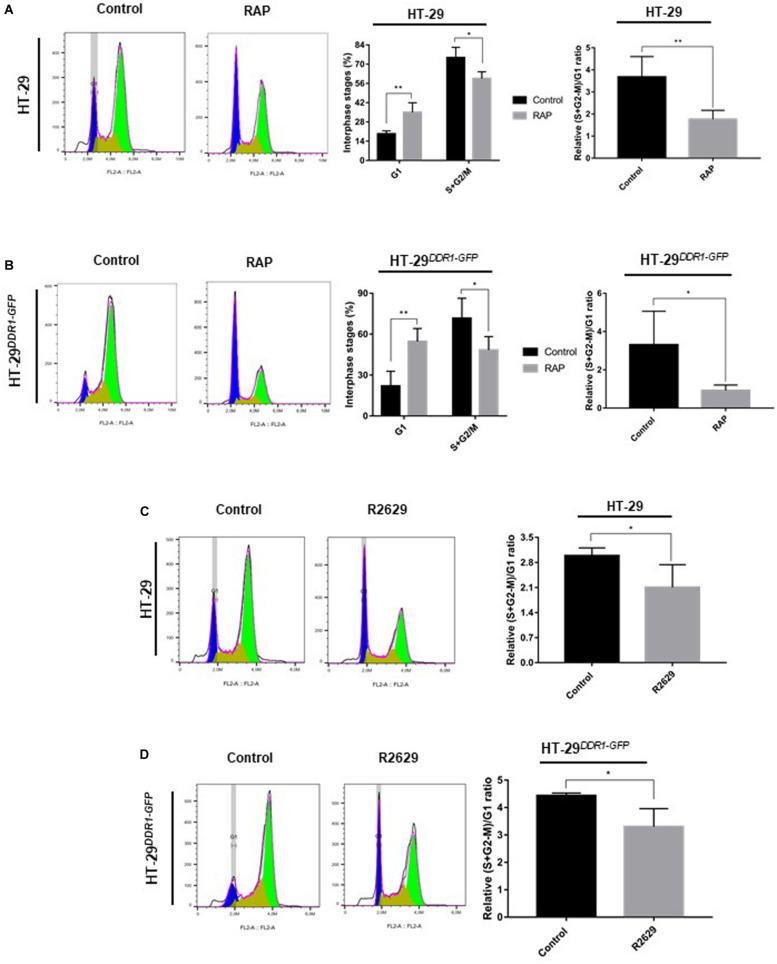
Inhibition of LRP-1-mediated endocytosis induces cell cycle arrest at G1 phase. HT-29 **(A,C)** and HT-29^*DDR*1–*GFP*^ **(B,D)** cells were grown on plastic surface and synchronized by double thymidine block. Synchronized cells were then seeded in 3D type I collagen matrix with or without 1 μM RAP **(A,B)** or LRP-1-blocking antibodies (R2629, 30 μg/mL) **(C,D)** for 24 h, followed by a cell cycle analysis. After nuclear staining with DAPI, 20.000 events were acquired and analyzed by flow cytometry. On the left colored panels, cell cycle distributions of HT-29 **(A,C)** and HT-29^*DDR*1–*GFP*^ **(B,D)** cells treated with or without RAP or R2629 for 24 h are shown as histogram plots of the FL3 fluorescence channel. On the right panels, histograms represent the percentage of interphase stages (G1, S+G2/M) and the relative (S+G2-M)/G1 ratio of HT-29 **(A,C)** or HT-29^*DDR*1–*GFP*^ **(B,D)** cells treated with (gray boxes) or without (black boxes) RAP or R2629. The data are presented as the mean SD. *p < 0.05; **p = 0.01, two samples *t*-test. Cell cycle assays were performed in four separate biological experiments for RAP treatment **(A,B)** and two separate experiments, each conducted in double triplicates for R2629 treatment **(C,D)**.

To further characterize the role of LRP-1 in the regulation of cancer cell proliferation, we investigated whether RAP treatment affects the cell cycle of HT-29 colon carcinomas. First, HT-29 and HT-29^*DDR*1–*GFP*^ cells were synchronized in G0/G1-phase by double thymidine blocking. The cells were then seeded in 3D collagen matrix to allow their re-entry into the cell cycle. The results of flow cytometric analysis revealed that HT-29 cells treated by RAP displayed an increased cell proportion in G1-phase (35% vs. 19%) and a decreased cell proportion in (S+G2-M)-phase (60% vs. 75%), compared to non-treated cells (Figure 5A). Moreover, the effect of RAP treatment on G1 and (S+G2-M)-phases was higher in HT-29^*DDR*1–*GFP*^ (54 and 48%, respectively) (Figure 5B). To confirm whether LRP-1 inhibition affects the G1/S transition, HT-29 and HT-29^*DDR*1–*GFP*^ were treated with the R2629 blocking antibody (Figures 5C,D). R2629 treatment has confirmed the obtained data wherein cells were treated with RAP. In fact, R2629-treated cells displayed also an increase in the proportion of cells in G1-phase and a decrease in S-phase cell population, compared to non-treated cells (Figures 5C,D).

### LRP-1 Counteracts the DDR1-Dependant Promotion of Apoptosis in Colon Carcinomas

**FIGURE 6:**
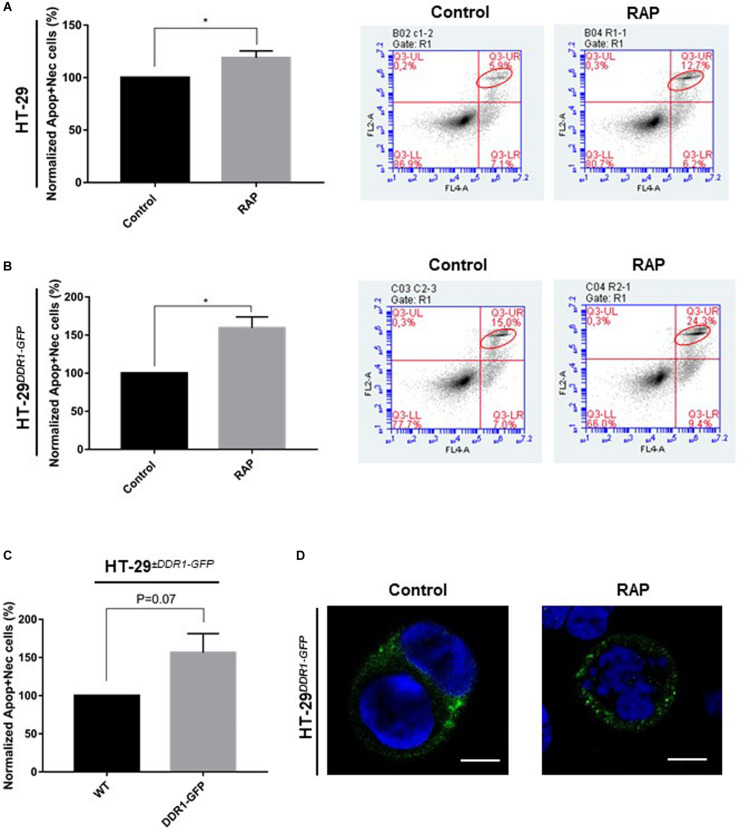
Inhibition of LRP-1 results in an increase in apoptosis. HT-29 **(A)** and, gray boxes HT-29^*DDR*1–*GFP*^ cells **(B)** were seeded in 3D type I collagen matrix and were treated without (black boxes) or with RAP (1 μM) for 3 days. The cells were then collected from digested matrix and suffered a rapid trypsinization before underwent an apoptotic assay. Apoptotic cells were stained with Annexin V and histogram (left panel), showed the percent of apoptotic. The values of treated samples were normalized to their controls, the data are represented as the mean SD, *p < 0.05, two sample *t*-test. **(C)** The plot represents the apoptotic indices of wild-type HT-29 overexpressing GFP (black boxes) and HT-29^*DDR*1–*GFP*^ (gray boxes) cells seeded in 3D collagen matrix. The apoptosis assays were performed in two distinct experiments, each done in double triplicates. **(D)** Immunostaining of recombinant DDR1 (green) in untreated (left picture) or RAP-treated (right picture) HT-29^*DDR*1–*GFP*^ cells. DNA is stained with DAPI. Scale bar: 5 μm.

The inhibition of breast cancer cell growth induced by type I collagen 3D matrices has been previously attributed to a strong DDR1-dependent induced apoptosis (Assent et al., 2015; Saby et al., 2018). To evaluate whether type I collagen/DDR1 axis can induce apoptosis in colon carcinoma, the apoptosis assay was performed using Annexin V staining and flow cytometry. As shown in Figures 6A,B, LRP-1 antagonization by RAP resulted in an increase in the proportion of apoptotic and necrotic cells in 3D collagen environment. Interestingly, this effect was higher in HT-29^*DDR*1–*GFP*^ cells (15.0% of apoptotic cells), compared to HT-29 cells (5.9% of apoptotic cells). The ability of DDR1 to increase apoptosis of colon carcinomas was confirmed in Figure 6C. These results were corroborated by immunofluorescence experiments. As shows in Figure 6D, the assay consistently shown the increased presence of nuclear condensation and DNA fragmentation upon LRP-1 inhibition.

## Discussion

The findings of this study have highlighted the first ever molecular association between LRP-1 and DDR1 in colon carcinoma. Indeed, we showed that the endocytic receptor LRP-1 established tight molecular connections with DDR1 at the plasma membrane of colon cancer cells. In this tumor context, we provide evidence that LRP-1 promotes cell proliferation through regulating the levels of membrane DDR1 in 3D collagen matrices. The LRP-1 mediated endocytosis of DDR1 supports colon carcinoma cell proliferation by promoting the entry of cell cycle to the S phase and decreasing apoptosis.

LRP-1 is considered as a key integrator of signals from the ECM and a multifunctional regulator of cancer-related events. Its overall function remains nevertheless extremely complex to decipher especially because the deregulation degree of its expression is highly variable depending on the type of tumors and the stage of cancer progression. In malignant diseases, the current trend seems to correlate LRP-1 overexpression with poor prognostic, increased cell proliferation, invasiveness and tumor recurrence (Catasus et al., 2011; Gheysarzadeh et al., 2019; Tian et al., 2019). To date, few studies have examined the contribution of LRP-1 in the field of CRC despite obvious clinical interest. We have recently highlighted that low LRP-1 immunohistochemistry score in malignant colon adenocarcinoma cells is a strong prognosis marker (Boulagnon-Rombi et al., 2018). We especially reported that in patients with metastases, LRP-1 expression predicts a shorter overall survival, especially when patients were treated by anti-VEGF therapies. The lower expression of LRP-1 in malignant cells is partly explained by LRP-1 gene mutation through the hypermutator type of CRC. In the present study conducted using relevant 3D collagen matrices, we showed in a surprising way that LRP-1 inhibition decreased colon carcinoma cell proliferation. Although these results seem to be conflicting with the previous data (Boulagnon-Rombi et al., 2018), it could be explained by the fact that the studied cell lines in this work are non-invasive cells in which LRP-1 expression is not modified and cell-surface DDR1 expression remains quite low due to the LRP-1-mediated internalization process, thus leading to high proliferation. In contrast, during metastasis development, we supposed that LRP-1 expression is down-regulated after cleavage by sheddases leading to a higher expression of DDR1 at the cell surface and then an increased tumor invasion. Although it is well documented that LRP-1 may activate crucial downstream signaling pathways such as Ras, c-Myc, MAPK, and Akt/PI3K, which are widely known as oncogenic pathways, especially in cell proliferation and survival processes (Van Gool et al., 2015), very few data have previously involved LRP-1 during cancer cell proliferation steps. Salama and collaborators reported the involvement of LRP-1:tPA pathway in promoting melanoma cell migration and proliferation (Salama et al., 2019). Their results, using loss- and gain-of-function strategy demonstrated a model wherein LRP-1 drives melanoma growth and metastases by enhancing ERK activation resulting in increased proteolytic events and in changing the cellular content within the tumor. Data from Beaujouin et al. (2010) also revealed that secreted pro-cath-D binds to LRP-1 promoting human mammary fibroblast outgrowth.

Interestingly, our findings stressed that LRP-1 displays a pro-proliferative effect on colon cancer cells only in 3D type I collagen matrices. During tumor progression, especially after degradation of the basement membrane, type I collagen is a key component of the stroma at the invasion front of human colorectal cancer (Brabletz et al., 2004). In addition to its properties as a scaffold protein, type I collagen can induce different cellular signaling pathways, which regulate several functions of tumor cells (Leitinger, 2011). Accumulating evidence suggest that DDR plays a key role in cancer progression by regulating the interactions of cells with the stromal collagen (Valiathan et al., 2012; Toy et al., 2015; Gao et al., 2016; Jeitany et al., 2018). Data obtained on HT-29 cells demonstrated that inhibition of LRP-1-dependent endocytosis by either RAP or R2629 antibodies led to membrane DDR1 accumulation in the same extent. We then demonstrated that LRP-1 and DDR1 are tightly associated in the same biomolecular complexes at the plasma membrane of colon carcinoma to constitute a new endocytosis complex. These results are even more interesting, as so far, little information is available concerning the regulation of DDR1 expression at the cell membrane. It is nevertheless known that activated DDR1 undergoes aggregation followed by cytoplasmic internalization and incorporation into early endosomes (Mihai et al., 2009). In mouse fibroblasts, DDR1 was reported to be internalized alone or complexed with other receptor tyrosine kinases (RTKs). Indeed, IGF-I receptor can phosphorylate DDR1 in breast carcinoma thus inducing co-internalization of the receptors and incorporation into early endosomes (Malaguarnera et al., 2015). Internalized RTKs can recycle back to the plasma membranes, be degraded, or undergo an endosome/Golgi/endoplasmic reticulum retrograde pathway. Interestingly, a novel mechanism whereby activated DDR1 plays a role of transcription factor has been demonstrated in injured human and mouse kidney proximal tubules (Chiusa et al., 2019).

Our findings showed that LRP-1 exerts its proliferative effects by down-regulating the amount of DDR1 at the plasma membrane. Indeed, by inducing the endocytosis of DDR1, LRP-1 counteracts the negative effect of DDR1 on cancer cell proliferation. Antagonization of LRP-1 by RAP or blocking antibodies indeed induced a significant cell cycle arrest in G1 phase, and this is magnified under DDR1 overexpression. Moreover, inhibition of LRP-1 by RAP treatment increases apoptosis of wild-type HT-29 cells and more importantly of HT-29^*DDR*1–*GFP*^. In a coherent way, overexpression of DDR1 in HT-29 cells favors cell cycle arrest and apoptosis of colon carcinoma in 3D environment. These data are consistent with those previously obtained by Erik Maquoi’s group demonstrating that MCF-7 and ZR-75-1 breast carcinoma cell growth was reduced in 3D type I collagen gels, but not when the cells were plated on a 2D matrix (Maquoi et al., 2012; Assent et al., 2015). Moreover, type I collagen was able to induce apoptosis in these cells. In fact, type I collagen can activate DDR1 to induce the expression of BIK, a pro-apoptotic member of the BCL-2 protein family, thereby triggering apoptotic cell death in these breast cancer cell lines (Assent et al., 2015). In addition, our group already demonstrated that young collagen inhibited cell proliferation and induced apoptosis when compared to the old one, due to a higher level of DDR1 phosphorylation (Assent et al., 2015; Saby et al., 2018). Furthermore, DDR2 is able to inhibit proliferation of human melanoma and fibrosarcoma cells by inducing a growth arrest in the G0/G1 phase of the cell cycle when the cells were plated on fibrillar collagen. This process was shown to be induced through p15INK4b cyclin-dependent kinase inhibitor, suggesting that this protein could be a downstream target of DDR2 signaling (Henriet et al., 2000; Wall et al., 2005, 2007). Moreover, DDR2, upon activation by 3D collagen, was able to target the cell cycle by increasing the expression of the cyclin-dependent kinase inhibitor p21^*CIP*1^ and thus inhibiting cell proliferation in a fibrosarcoma model (Saby et al., 2016). In contrast, DDR1 activation can also induce pro-survival signals (Ongusaha et al., 2003). In colon carcinoma cells, DDR1 regulates the cleavage of Notch 1 by a γ-secretase and the subsequent release and translocation of its intracellular domain to the nucleus to stimulate pro-survival genes (Kim et al., 2011). The collective findings suggest that DDR1 can induce survival as well as apoptosis, highly depending on experimental settings.

Finally, we identified a new molecular way that controls the cell-surface expression of DDR1 and suggested an additional role of LRP-1 as a key sensor of the tumor microenvironment.

## Data Availability Statement

All datasets generated for this study are included in the article/Supplementary Material.

## Author Contributions

CL was responsible for the execution of experiments, data analysis, and preparation of the manuscript. CH, GC, AB, and VL supported the experimental work and data analysis. AB contributed to the interpretation of the results and the writing of the manuscript. AA-C, HM, and SD designed, supervised the study, and wrote the manuscript. All authors critically commented on and approved the final submitted version of the manuscript.

## Conflict of Interest

The authors declare that the research was conducted in the absence of any commercial or financial relationships that could be construed as a potential conflict of interest.

## Supplementary Material

FIGURE S1 No effect of IgG control on colorectal cancer cell proliferation n 3D matrix. Colorectal carcinomas were cultured in 3D type I collagen matrices without (black boxes) or with non-reactive IgG (light gray boxes) treatment. After 5 days of culture, cell growth indices were assessed using at least three separate sets of culture, all conditions were repeated at least three times. ns: not significant.

FIGURE S2 Effect of LRP-1 antagonist on RKO cell proliferation. **(A)** Whole cell extracts from RKO cells were analyzed by SDS PAGE followed by western blotting using anti-DDR1 antibodies. **(B)** RKO cells were cultured in 2D type I collagen coating (left panel) or 3D type I collagen matrices (right panel) without (black boxes) or with RAP (500 nM, light gray boxes) treatment. After 5 days of culture, cell growth indices were assessed using at least three separate sets of culture, all conditions were repeated at least three times. ns: not significant.
