# Supplementary figures and images for: LRP-1 Promotes Colon Cancer Cell Proliferation in 3D Collagen Matrices by Mediating DDR1 Endocytosis
Source: Front Cell Dev Biol. 2020 Jun 3;8:412. doi: 10.3389/fcell.2020.00412 (PMC7283560; doi:10.3389/fcell.2020.00412)

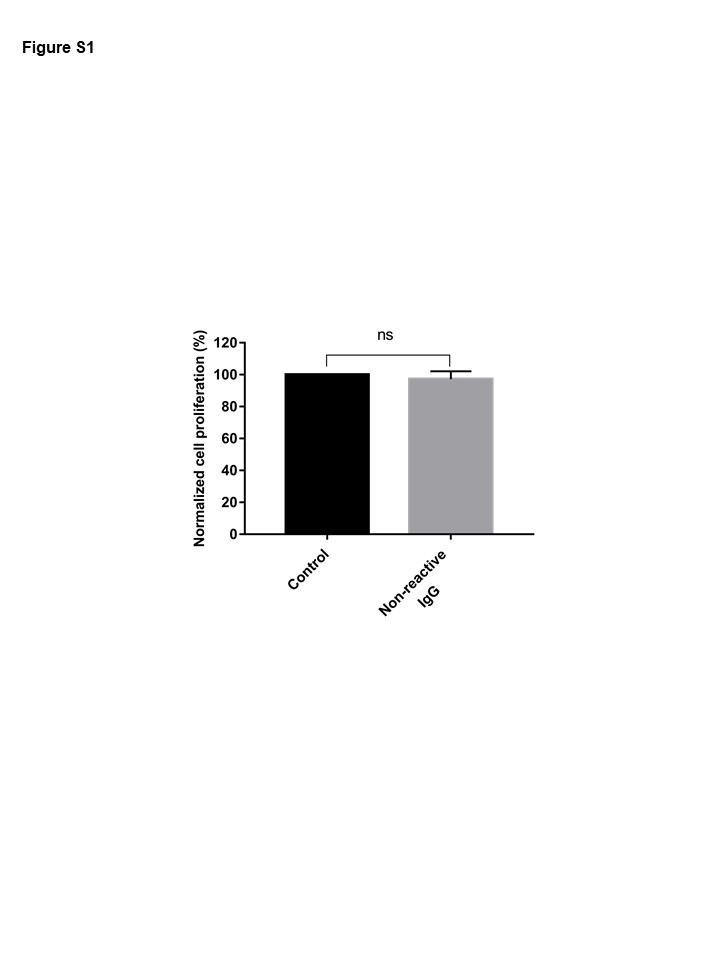

Supplement: FIGURE S1 — No effect of IgG control on colorectal cancer cell proliferation n 3D matrix. Colorectal carcinomas were cultured in 3D type I collagen matrices without (black boxes) or with non-reactive IgG (light gray boxes) treatment. After 5 days of culture, cell growth indices were assessed using at least three separate sets of culture, all conditions were repeated at least three times. ns: not significant. [file Image_1.jpeg]

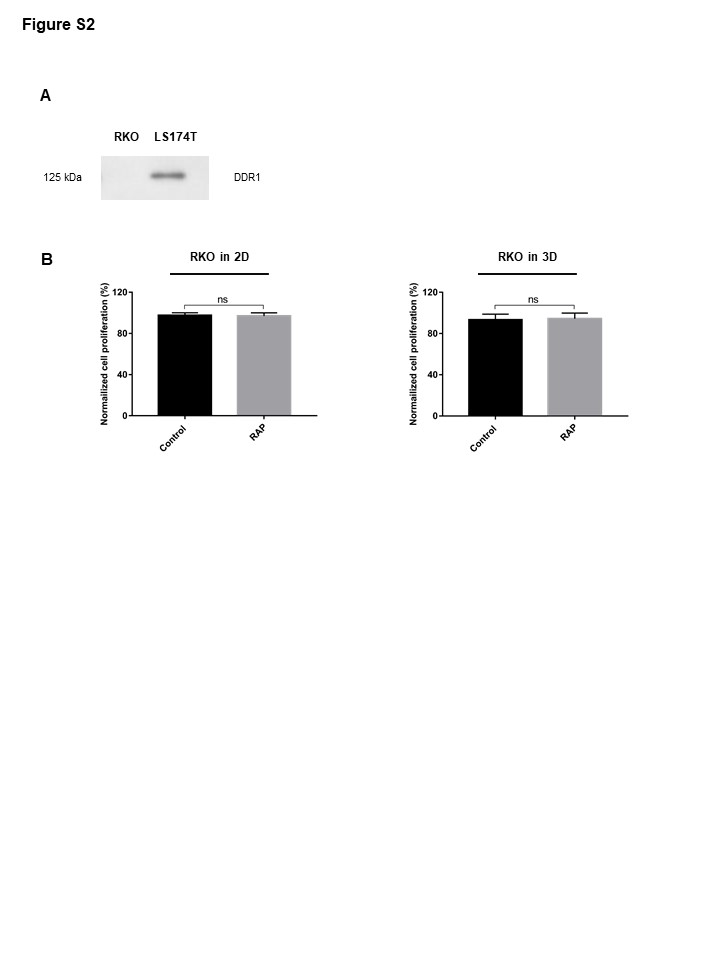

Supplement: FIGURE S2 — Effect of LRP-1 antagonist on RKO cell proliferation. (A) Whole cell extracts from RKO cells were analyzed by SDS PAGE followed by western blotting using anti-DDR1 antibodies. (B) RKO cells were cultured in 2D type I collagen coating (left panel) or 3D type I collagen matrices (right panel) without (black boxes) or with RAP (500 nM, light gray boxes) treatment. After 5 days of culture, cell growth indices were assessed using at least three separate sets of culture, all conditions were repeated at least three times. ns: not significant. [file Image_2.jpeg]
